# Electrical Properties and Interfacial Studies of Hf*_x_*Ti_1–*x*_O_2_ High Permittivity Gate Insulators Deposited on Germanium Substrates

**DOI:** 10.3390/ma8125454

**Published:** 2015-12-02

**Authors:** Qifeng Lu, Yifei Mu, Joseph W. Roberts, Mohammed Althobaiti, Vinod R. Dhanak, Jingjin Wu, Chun Zhao, Ce Zhou Zhao, Qian Zhang, Li Yang, Ivona Z. Mitrovic, Stephen Taylor, Paul R. Chalker

**Affiliations:** 1 Department of Electrical Engineering and Electronics, University of Liverpool, Liverpool L69 3GJ, UK; qifeng@liverpool.ac.uk (Q.L.); Y.Mu@student.liverpool.ac.uk (Y.M.); jingjin.wu@liverpool.ac.uk (J.W.); ivona@liverpool.ac.uk (I.Z.M.); s.taylor@liverpool.ac.uk (S.T.); 2 Center for Materials and Structures, School of Engineering, University of Liverpool, Liverpool L69 3GH, UK; eg0u5124@liverpool.ac.uk (J.W.R.); pchalker@liverpool.ac.uk (P.R.C.); 3 Department of Physics, University of Liverpool, Liverpool L69 7ZE, UK; M.Althobaiti@liverpool.ac.uk (M.A.); vin@liverpool.ac.uk (V.R.D.); 4 Nano and Advanced Materials Institute, Hong Kong University of Science and Technology, Kowloon 999077, Hong Kong, China; garyzhao@ust.hk; 5 Department of Electrical and Electronic Engineering, Xi’an Jiaotong-Liverpool University, Suzhou 215123, China; 6 Department of Chemistry, Xi’an Jiaotong-Liverpool University, Suzhou 215123, China; Qian.Zhang@xjtlu.edu.cn (Q.Z.); li.yang@xjtlu.edu.cn (L.Y.)

**Keywords:** Ge substrate, titanium-doped hafnium oxide, XPS, XRD, AFM

## Abstract

In this research, the hafnium titanate oxide thin films, Ti*_x_*Hf_1–*x*_O_2_, with titanium contents of *x* = 0, 0.25, 0.9, and 1 were deposited on germanium substrates by atomic layer deposition (ALD) at 300 °C. The approximate deposition rates of 0.2 Å and 0.17 Å per cycle were obtained for titanium oxide and hafnium oxide, respectively. X-ray Photoelectron Spectroscopy (XPS) indicates the formation of GeO*_x_* and germanate at the interface. X-ray diffraction (XRD) indicates that all the thin films remain amorphous for this deposition condition. The surface roughness was analyzed using an atomic force microscope (AFM) for each sample. The electrical characterization shows very low hysteresis between ramp up and ramp down of the Capacitance-Voltage (CV) and the curves are indicative of low trap densities. A relatively large leakage current is observed and the lowest leakage current among the four samples is about 1 mA/cm^2^ at a bias of 0.5 V for a Ti_0.9_Hf_0.1_O_2_ sample. The large leakage current is partially attributed to the deterioration of the interface between Ge and Ti*_x_*Hf_1–*x*_O_2_ caused by the oxidation source from HfO_2_. Consideration of the energy band diagrams for the different materials systems also provides a possible explanation for the observed leakage current behavior.

## 1. Introduction

Recently, germanium has emerged as a promising candidate for a channel material to be used in high-speed metal-oxide-semiconductor (MOS) devices, mainly due to germanium’s high carrier mobility (approximately ×2 for electrons and ×4 for holes compared with those of silicon) [1,2,3,4,5]. However, due to the lack of stable native oxide of germanium, it was difficult to fabricate a Ge Metal-Oxide-Semiconductor Field Effect Transistor (MOSFET) and a variety of dielectric materials were attempted. Among the various candidates, hafnium-based gate stacks, such as HfO_2_, HfON, and LaHfO*_x_*, have proven to be possible solutions for Ge MOS devices and transistors due to their relatively good reliability and high performance [6,7,8,9]. However, the reported dielectric constants of hafnium-based gate stacks varied from 11.5 to 21, which limited further scaling into the sub-nanometer regime [10,11]. In order to overcome this problem, a number of trials were carried out to further increase the permittivity of the dielectrics. One approach was to add a smaller amount of rare earth materials to the oxides to stabilize the crystal phase with a higher relative dielectric constant, such as lanthanum doped zirconium oxide [12,13]. Similar trials were performed on the hafnium oxide deposited on the silicon substrates, although the increase in the dielectric constant was not significant [14,15]. Another possible solution was to mix hafnium oxide with other dielectric materials with higher permittivity, such as titanium oxide (with *k* ~ 50–80). The high dielectric constant of the titanium oxide originates from the soft phonons of titanium, and an increase in the overall dielectric constant of gate oxides after mixing HfO_2_ and TiO_2_ was achieved [16,17]. Although the addition of TiO_2_ improved the dielectric constant of an HfO_2_-based material, the small energy band gap of TiO_2_ [18], which would result in a large leakage current, remained an issue to be considered [17]. Thus, the influence of different amounts of titanium oxide on the properties of the HfO_2_-based material is of great interest. In addition, the deterioration of the interface due to the oxidation source borne by the high-κ materials was observed, and the effective passivation of the germanium surface is still an open question [3]. In order to minimize the deterioration of the interface and suppress the growth of the unstable native oxide of germanium, a number of methods have been conceived to passivate the germanium surface, such as NH_3_ and sulfur treatment [19,20], or inserting an interfacial layer, such as aluminum oxide [21] between the high-κ thin film and the germanium substrate. 

In this work, a 0.3 nm Al_2_O_3_ interfacial layer was deposited on the germanium substrate by atomic layer deposition (ALD) to passivate the surface. Subsequently, the thin films with different content levels of the TiO_2_ in HfO_2_ were deposited by ALD. The effect of TiO_2_ content in hafnium oxide was explored in terms of physical and electrical properties. Furthermore, the interface quality and chemical structure between the oxides and substrates were investigated. The results of the measurements and the performance of the thin films of TiO_2_-HfO_2_ deposited on Ge substrate are presented and discussed in this paper.

## 2. Results and Discussion

**Figure 1:**
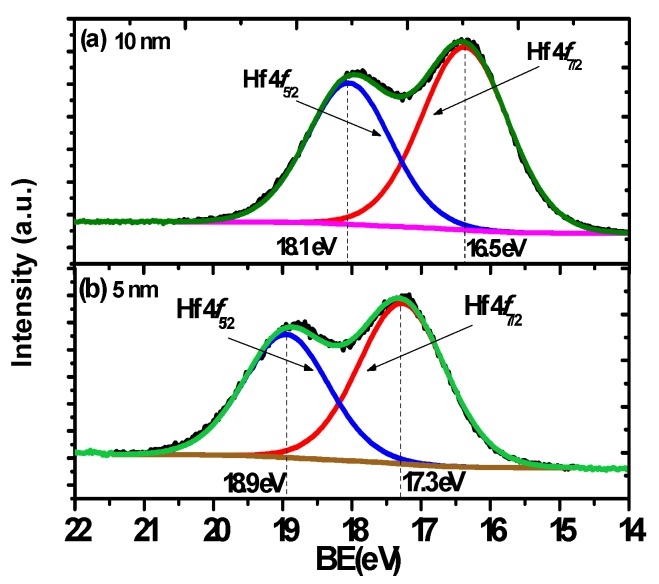
The XPS line shape for HfO_2_ thin films with the thickness of (**a**) 10 nm and (**b**) 5 nm. The sample with a thickness of 10 nm has the lower bonded peak at the position of 16.5 eV for Hf 4*f*_7/2_ spectra. For the sample with a thickness of 5 nm, the bonded peak for Hf 4*f*_7/2_ spectra is centered at 17.3 eV, with a difference of 0.8 eV in comparison to 10 nm one. This shift is probably due to the reaction of HfO_2_ with the germanium for the 5 nm HfO_2_ sample.

**Figure 2:**
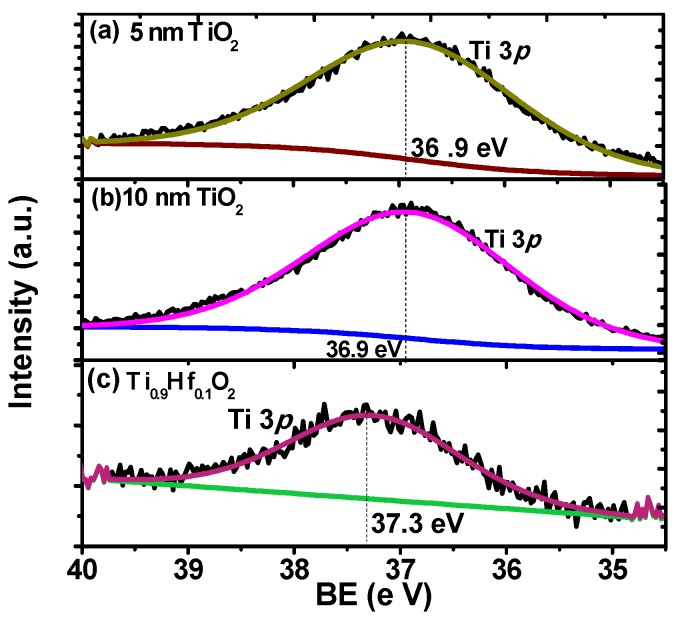
The Ti 3*p* spectra from: (**a**) 5 nm TiO_2_; (**b**) 10 nm TiO_2_; and (**c**) 5 nm Ti_0.9_Hf_0.1_O_2_ thin films. The 5 nm and 10 nm thickness TiO_2_ samples share the same Ti 3*p*_3/2_ binding energy centered at the 36.9 eV. A small difference, 0.4 eV, is observed in binding energy of Ti 3*p* spectra between TiO_2_ at 36.9 eV and in the Ti_0.9_Hf_0.1_O_2_ samples at 37.3 eV.

X-ray Photoelectron Spectroscopy (XPS) was used to characterize the quality of the interface and the thin films in the stacks. Firstly, the XPS was performed on the 5 nm and 10 nm HfO_2_ thin films to find out the chemical structure of the HfO_2_ samples in the depth direction. XPS is a surface sensitive technique so the interface was probed by using a 5 nm nominal thickness film on the germanium substrate. As shown in Figure 1a,b, the Hf 4*f* line shape is typically composed of a 4*f*_5/2_ and 4*f*_7/2_ spin-orbit doublet [22]. With respect to the Hf 4*f_7/2_* peak positions, there is a clear difference between the two thin films with different thicknesses. The sample with a thickness of 10 nm has the lower binding energy (BE) peak at the position of 16.5 eV, which is tentatively assigned to stoichiometric HfO_2_. For the sample with a thickness of 5 nm, the binding energy of the peak is centered at 17.3 eV, a difference of 0.8 eV in comparison to the 10 nm one. This shift is indicative of the greater interaction between the HfO_2_ and Ge, and suggests stoichiometric and chemical changes at the interface. This is in accord with previous research, which has reported that the binding energy of Hf 4*f_7/2_* peak in HfSi*_x_*O*_y_* was 1 eV higher than that from HfO_2_, which has a binding energy in the range of 16.5–17 eV [23,24]. Similar results have also been found for the Ge MOS device, which stated that about a 0.5 eV shift of binding energy existed for the Hf 4*f_7/2_* peak from HfGeO*_x_* compared with that from HfO_2_ [25,26]. We can thus tentatively assign the shift in the Hf 4*f* binding energy to the formation of a germinate, HfGeO*_x_*. In contrast, the XPS results in Figure 2a,b for the TiO_2_ samples in this experiment show that the Ti 3*p*_3/2_ peaks for the 5 nm and 10 nm thickness samples are centered at the same position with binding energy of 36.9 eV, suggesting that no chemical structure change occurs for the TiO_2_ samples in depth direction. Based upon the above analysis, it is inferred that HfO_2_ will react with the Ge atoms at the interface without an effective passivation of the substrate. Formation of HfGeO*_x_* at the interface deteriorates the interface and possibly increases the leakage current in the stack [26].

**Figure 3:**
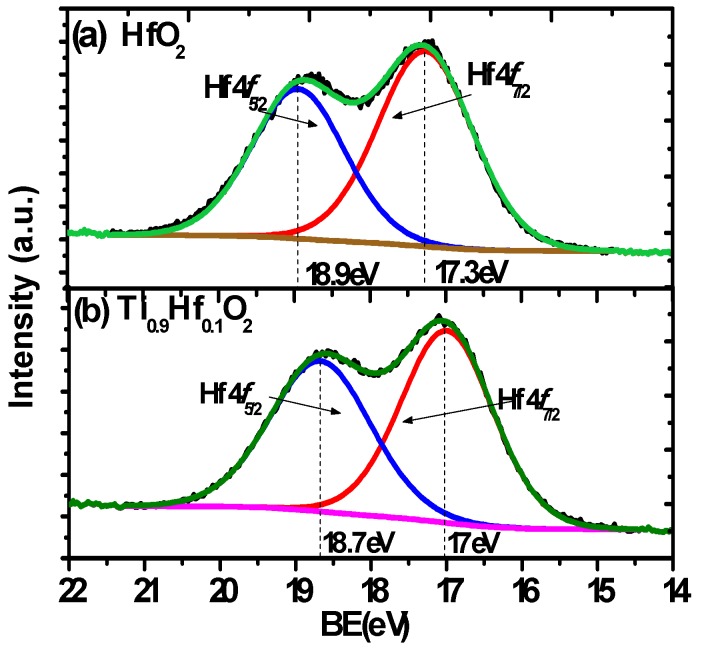
The Hf 4*f* spectra from 5 nm thick films of (**a**) HfO_2_ and (**b**) Ti_0.9_Hf_0.1_O_2_ on Ge. The Hf 4*f*_7/2_ binding energy from Ti_0.9_Hf_0.1_O_2_ is 17 eV with a smaller shift compared to the HfO_2_ with a shift of 17.3 eV.

Figure 2c compares the Ti 3*p* spectrum from the Ti_0.9_Hf_0.1_O_2_ sample with the Ti 3*p* from a pure TiO_2_ film on germanium, while Figure 3 shows the Hf 4*f*_7/2_ spectra from the same 5 nm thick Ti_0.9_Hf_0.1_O_2_ sample and compares it to Hf 4*f*_7/2_ from a pure HfO_2_ film. It is clear that the Hf 4*f*_7/2_ binding energy from Ti_0.9_Hf_0.1_O_2_, 17 eV, has a smaller difference in comparison to the pure HfO_2_ at 17.3 eV (Ti_0.25_Hf_0.75_O_2_ has the same Hf 4*f*_7/2_ binding energy as Ti_0.9_Hf_0.1_O_2_, 17 eV, not shown here). In addition, Figure 2a,c show that there is also a difference of 0.4 eV in the binding energy of the Ti 3*p* spectra between TiO_2_ (36.9 eV) and Ti_0.9_Hf_0.1_O_2_ (37.3 eV). For the Ti_0.25_Hf_0.75_O_2_ sample, the Ti 3*p* spectrum was found shifted to a higher binding energy by about 0.2 eV, while Hf 4*f* shifted to lower binding energy by about 0.3 eV. The shift of the Hf 4*f*_7/2_ peak in the Ti*_x_*Hf_1–*x*_O_2_ samples to a lower binding energy and the Ti 3*p* to a higher binding energy suggests that an electron transfer from HfO_2_ to TiO_2_ takes place as a result of chemical mixing between TiO_2_ and HfO_2_.

**Figure 4:**
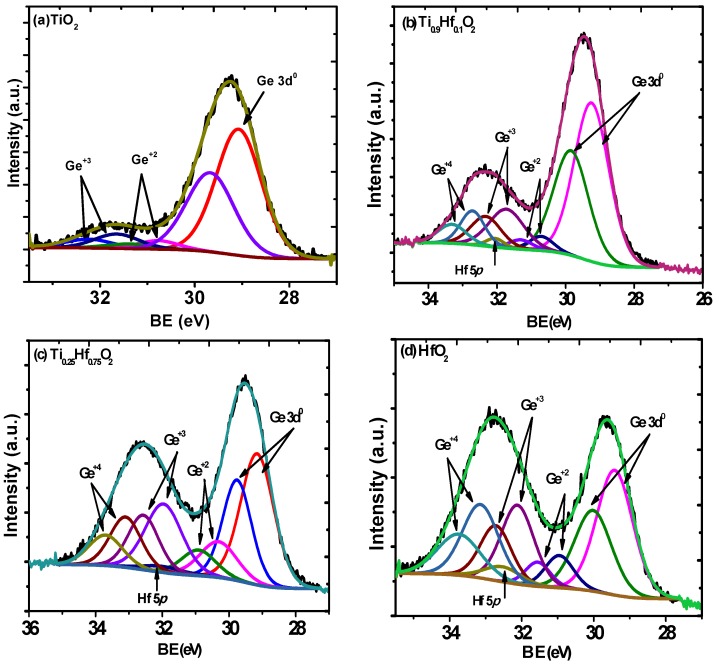
Ge 3*d* spectra from 5 nm thin films of: (**a**) TiO_2_; (**b**) Ti_0.9_Hf_0.1_O_2_; (**c**) Ti_0.25_Hf_0.75_O_2_; and (**d**) HfO_2_ samples. The presence of Ge^+2^, Ge^+3^, and Ge^+4^ is due to the Ge oxidation at the interfacial region. There is an increment of GeO*_x_* peak intensity, especially for Ge^+4^ peaks, with the increase of the HfO_2_ concentration.

**Figure 5:**
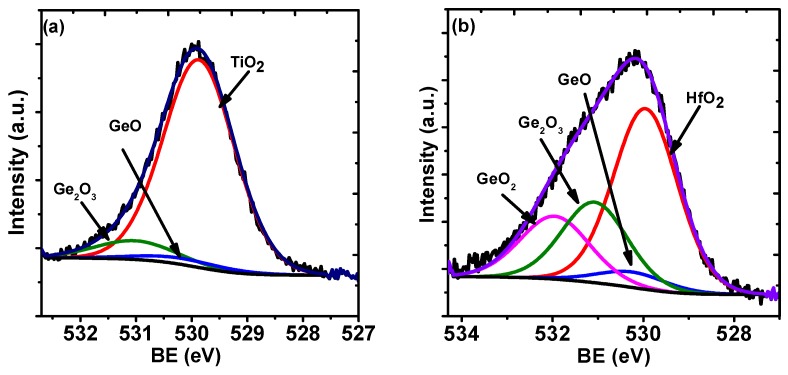

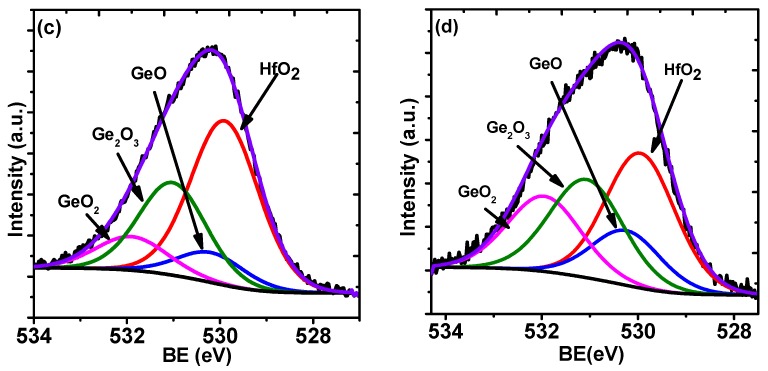
O 1s spectra from 5 nm films of: (**a**) TiO_2_; (**b**) Ti_0.9_Hf_0.1_O_2_; (**c**) Ti_0.25_Hf_0.75_O_2_; and (**d**) HfO_2._

In addition, from the analysis of the Ge 3*d* spectra from the four samples with a thickness of 5 nm shown in Figure 4, more information about the Ge surface can be deduced. The corresponding O 1*s* spectra from the four samples are shown in Figure 5. The peaks corresponding to Ge from elemental Ge and GeO*_x_* are labeled in the figures. The presence of Ge^2+^, Ge^3+^, and Ge^4+^ is due to the oxidation of the germanium substrate at the interfacial region, as well as possible germinate formation. Table 1 shows the compositions extracted from the line fits, relative to the bulk substrate Ge^0^ peak, of the various components at the interface for the four samples. It is clear that the oxidation is much less in the samples with TiO_2_ (Figure 4a) compared with the other samples, while the oxidation of the substrate in the case of HfO_2_ is much greater (Figure 4d). The fitting of the spectra shows an absence of Ge^+4^ in the TiO_2_ sample, while an incremental increase of the GeO*_x_* intensity, especially Ge^+4^, is observed with the increasing HfO_2_ content. This suggests that increasing the amount of HfO_2_ in the dielectric films provides more oxidation sources to the interface [27]. This has also been observed in other research, which states that Ge atoms were oxidized by the oxygen atoms provided by the HfO_2_ layer [3]. Furthermore, the Hf 4*f*_7/2_ binding energy difference for the HfO_2_ samples with different thickness, shown in Figure 1a,b and discussed above, also supports this finding. Therefore, it is inferred that the HfO_2_ is a factor in the oxidation and has a deteriorating effect on the interface. With regard to the significant binding energy shift of the Ge 3*d* spectra from the samples, it is partially attributed to the presence of a mixture of oxides of GeO*_x_* (where *x* < 2) and GeO_2_ at the interfacial region. The GeO*_x_*, referred to as a suboxide in the following discussion, consists of a structure with less than four oxygen atoms attached to one Ge. GeO is known to exhibit a signal at a binding energy lower than that of GeO_2_ [28]. In this experiment, the concentration of GeO_2_ and the *x* value differ from each other for the four samples. For Ge^4+^, it is absent in the pure TiO_2_ layer on the germanium, and it is minimal for the Ti_0.9_Hf_0.1_O_2_, while it is at a maximum for the pure HfO_2_ layer. Therefore, this behavior causes the resultant intensity of the spectral component due to GeO_2_ increasing, with a concomitant decrease in intensity of the peak of GeO*_x_* for oxygen rich samples, and this intensity variation leads to a shift of the overall peak [28,29]. A similar phenomenon of Ge 3*d* spectra shift was also observed by Caymax in the interface study of the HfO_2_ gate dielectric deposited on Ge [30]. In addition, the formation of HfGeO*_x_* due to the reaction between HfO_2_ and Ge is also a possible contributing factor to the shift of the Ge 3*d* spectra [25,31]. 

**Table 1:** Compositions extracted from the line fits shown in Figure 4, relative to the bulk substrate Ge^0^ peak for the four samples.

Materials	Ge^+2^	Ge^+3^	Ge^+4^
TiO_2_	0.06	0.12	–
Ti_0.9_Hf _0.1_O_2_	0.06	0.24	0.14
Ti_0.25_Hf_0.75_O_2_	0.17	0.24	0.41
HfO_2_	0.13	0.45	0.75

**Figure 6:**
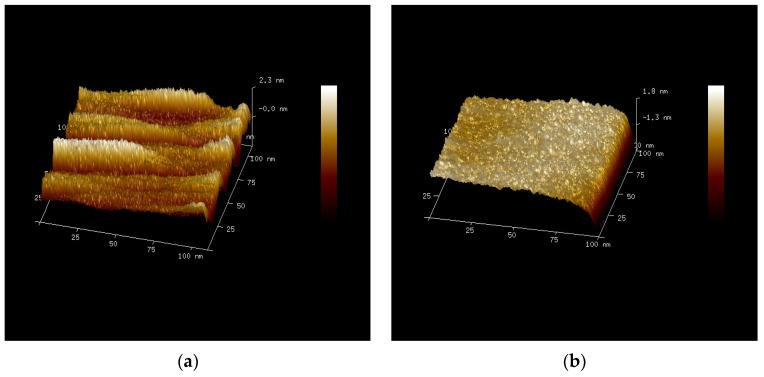

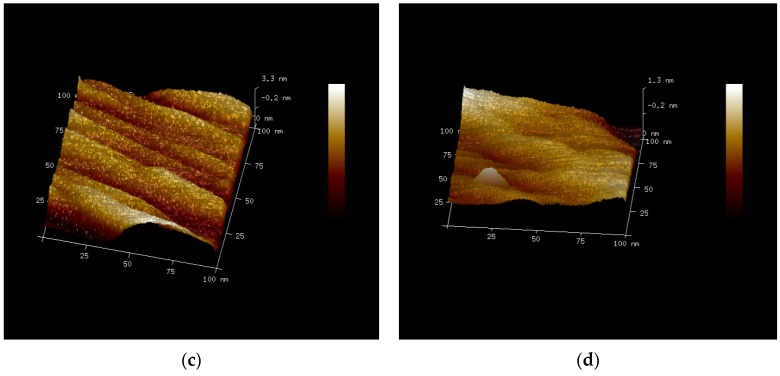
Atomic force microscope (AFM) images of the samples: (**a**) TiO_2_; (**b**) Ti_0.9_Hf_0.1_O_2_; (**c**) Ti_0.25_Hf_0.75_O_2_; and (**d**) HfO_2_. The roughness (*R*_rms_) for each sample is 0.325 nm, 0.431 nm, 0.425 nm, and 0.202 nm.

An atomic force microscope (AFM) was used to examine the surface roughness of the samples and the results for a scan area of 100 nm × 100 nm are presented in Figure 6. The surface roughness of the samples is quantitatively determined by the root-mean-squared roughness (*R*_rms_), defined as

(1) $$R_{\text{rms}}=\sqrt{\frac{\sum_{n=1}^{N}{\left(z_{\text{n}}-\bar{z}\right)}^{2}}{N-1}}$$

where *z*_n_ is the measured height, $\bar{z}$ is the average height of the sample, and *N* is the number of measurements.

As can be seen, all the samples exhibit good surface morphology with a roughness *R*_rms_ of 0.325 nm, 0.431 nm, 0.425 nm, and 0.202 nm for TiO_2_, Ti_0.9_Hf_0.1_O_2_, Ti_0.25_Hf_0.75_O_2,_ and HfO_2_ respectively. The roughness of the thin films less than 0.5 nm demonstrates a nearly atomically smooth surface [32].

**Figure 7:**
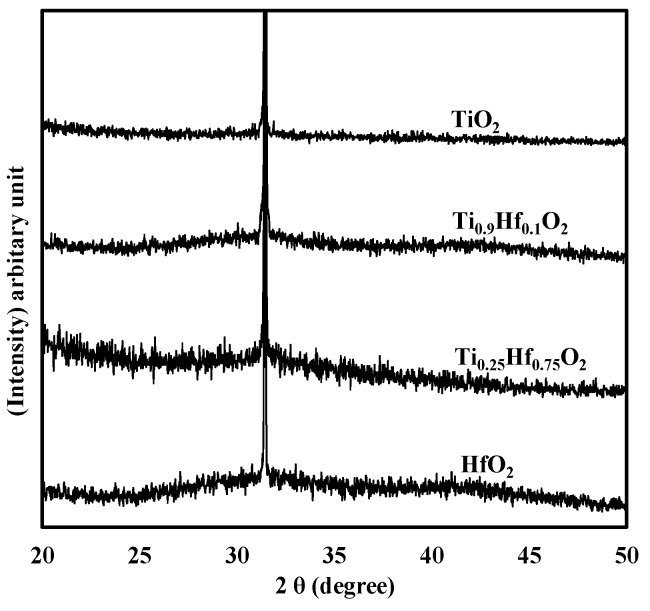
XRD patterns for the 10 nm HfO_2_, Ti_0.25_Hf_0.75_O_2_, Ti_0.9_Hf_0.1_O_2_, and TiO_2_ thin films deposited on the germanium substrate. No noticeable diffraction peaks are observed, except for the one from the substrate.

Figure 7 shows the XRD patterns for the four samples with different compositions of TiO_2_ and HfO_2_. The measurement was performed on the samples with a nominal thickness of 10 nm (the actual thickness was in the range 8 to 11 nm determined by ellipsometer). For all the samples, no noticeable diffraction peaks are observed, except for the one coming from the substrate centered at around 31.5°. According to the results of the XRD patterns only, it seems that all the thin films remained amorphous under these deposition conditions. However, due to the small thickness of the thin films, around 10 nm, the sensitivity of the XRD is probably not sufficient to detect a limited amount of the crystalline phase if it is present in the thin films, as has been previously pointed out [33,34]. It is possible that TEM (or SEAD) could further prove the exact morphology of the thin films.

**Figure 8:**
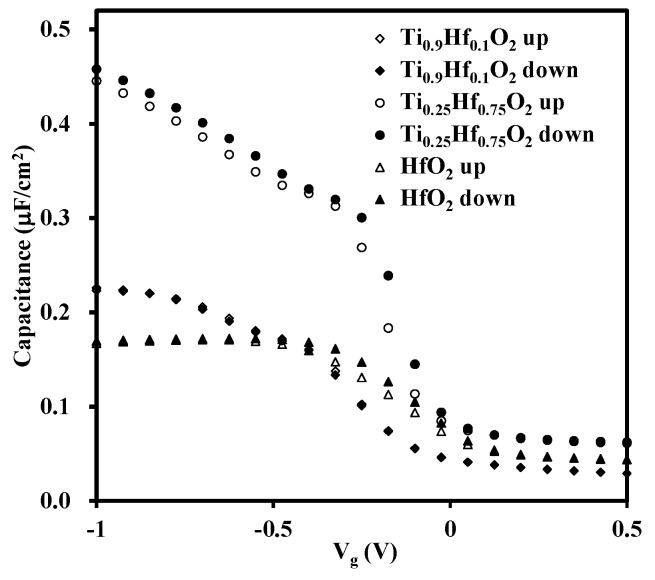
CV characteristics for the three samples of Ti_0.9_Hf_0.1_O_2_, Ti_0.25_Hf_0.75_O_2_, and HfO_2_. The gate voltage was swept from −1 V to 0.5 V at a frequency of 1 MHz, and no horizontal shift is observed. For the CV characteristics of the Ti_0.25_Hf_0.75_O_2_ sample, saturation in the accumulation region is not obtained regardless of the bias voltage level, which is attributed to the large leakage current observed in this sample.

The Capacitance-Voltage (CV) curves were obtained by sweeping the gate voltage from −1 V to 0.5 V in both directions (ramp up and ramp down) at a frequency of 1 MHz using an Agilent 4284A LCR meter (Agilent, Santa, CA, USA). Due to an unacceptable distortion of the CV characteristics caused by a large leakage current for the TiO_2_ sample, reported below, only the CV curves extracted from HfO_2_, Ti_0.25_Hf_0.75_O_2_, and Ti_0.9_Hf_0.1_O_2_ samples are presented in Figure 8. The high frequency CV measurements on the three as-grown thin films show that the samples have low trap densities because there is almost no hysteresis between the ramp up and ramp down of the CV curves. Due to the dielectric relaxation, likely caused by parasitic effect, lossy interfacial layer, and surface roughness of the samples, the obvious frequency dispersion of the samples is observed. Therefore, the vertical change of the CV characteristics is not discussed in this paper [35,36,37]. Regarding the CV characteristics of the Ti_0.25_Hf_0.75_O_2_ sample, it is noted that saturation in the accumulation region is not obtained, regardless of the bias voltage level. This behavior is attributed to the large leakage current for this sample, which is possibly partially related to the deterioration of the interface as discussed above in the section for XPS analysis. Further comments regarding the leakage current are made in the following section.

**Figure 9:**
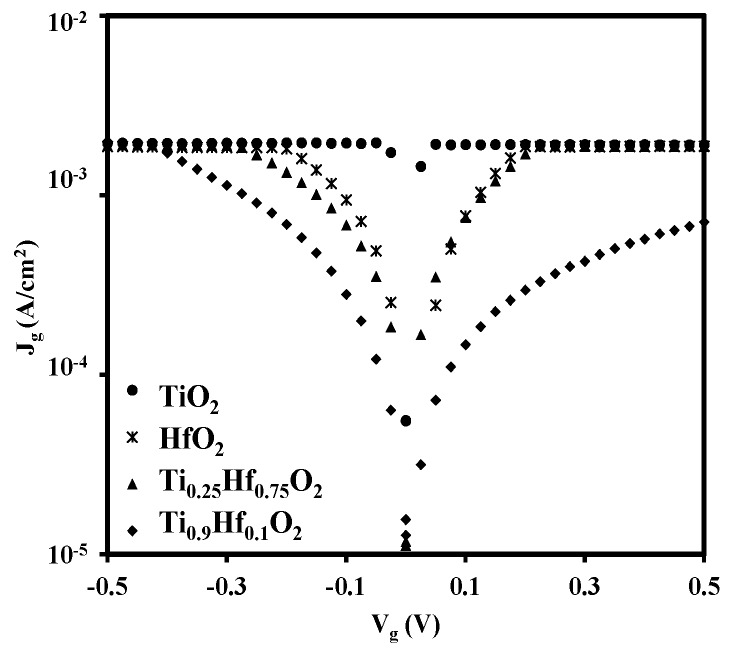
Gate leakage current density (*J*_g_) *versus* gate voltage (*V*_g_) for samples of HfO_2_, Ti_0.25_Hf_0.75_O_2_, Ti_0.9_Hf_0.1_O_2_, and TiO_2_. The titanium oxide occupies the highest leakage current level followed by hafnium oxide and Ti_0.25_Hf_0.75_O_2_ thin films with similar leakage current levels. The Ti_0.9_Hf_0.1_O_2_ sample has the lowest leakage current.

**Figure 10:**
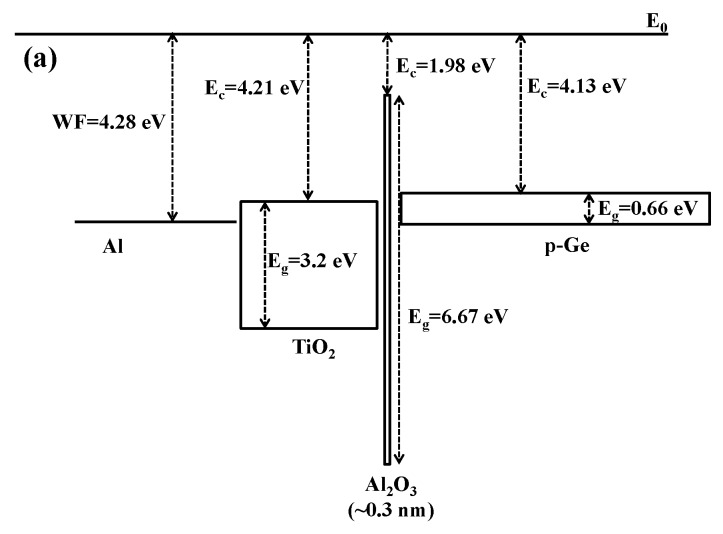

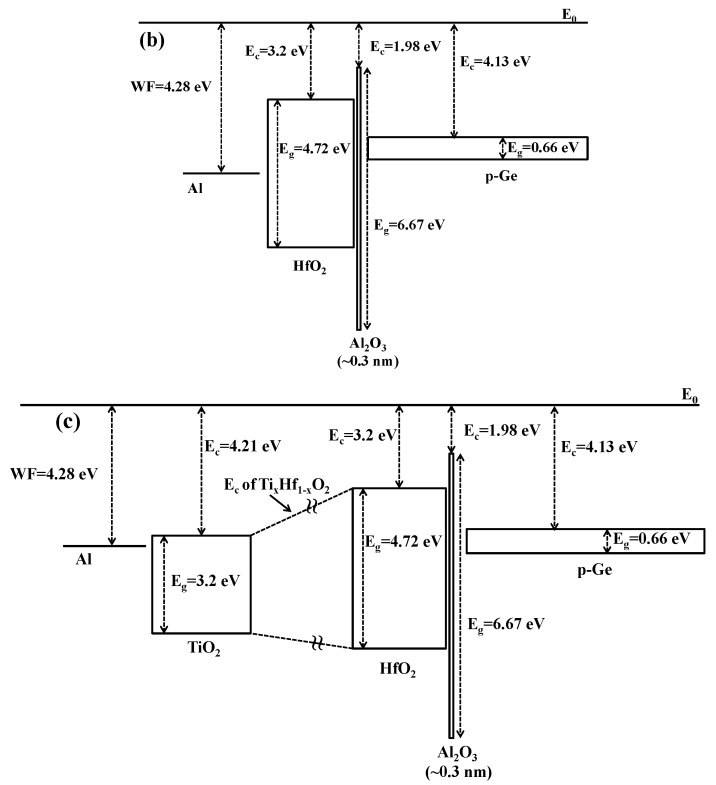
Energy band diagrams for: (**a**) titanium oxide; (**b**) hafnium oxide; and (**c**) titanium doped hafnium oxide. The work function of Al is represented by WF. Titanium oxide has relativly small band gap (3.2 eV) and the conduction band minimum is 4.21 eV below the vacuum level. Modest voltages applied to the gate can result in large leakage currents. The incorporation of hafnium oxide results in a significantly wider band gap and a lower conduction band minimum compared to that of TiO_2_. Therefore, the resulting leakage current of HfO_2_ is 5 times lower than that of TiO_2_ for the same applied gate voltage, regardless of the interfacial deterioration, as shown in Figure 9.

Figure 9 illustrates the relationship between the gate leakage current density (*J*_g_) and the bias voltage (*V*_g_) of the samples. The maximum current limit on our instrument was set at 2 mA. From observations drawn from Figure 9, it is apparent that the titanium oxide has the highest leakage current level, followed by hafnium oxide and Ti_0.25_Hf_0.75_O_2_ thin films, both of which have similar leakage current levels. The Ti_0.9_Hf_0.1_O_2_ sample has the lowest leakage current, with less than 1 mA/cm^2^ at a bias voltage of 0.5 V. The large leakage current for the TiO_2_ sample is attributed to the small band gap of TiO_2_ as shown in Figure 10a and discussed further below. For the Ti_0.9_Hf_0.1_O_2_, Ti_0.25_Hf_0.75_O_2_, and HfO_2_ samples with a larger band gap, it is clear that the leakage current increases with greater amounts of HfO_2_. Previous research has also reported that a large leakage current was caused by the formation of HfGeO*_x_* at the interface between HfO_2_ and Ge, and the leakage current was reduced if a germanium nitride barrier layer was first introduced, preventing the formation of HfGeO*_x_* [3]. High leakage current behavior, therefore, is probably due to the deterioration of the interfacial layer caused by the interaction of HfO_2_ and Ge, which is consistent with the results shown in Figure 4. Thus, the increase in the leakage current clearly correlates with the hafnium oxide rich samples. For the TiO_2_ doped samples, the TiO_2_ would react with HfO_2_ to form HfTiO*_x_*, consuming the HfO_2_, which would otherwise have reacted with the Ge at the interface. It is also possible that other mechanisms may also exist to suppress the leakage current, as has been observed in the titanium doped tantalum oxide. Titanium doping was found to suppress the oxygen vacancies in tantalum oxide capacitors, which resulted in a significant reduction in the leakage current [38]. For the HfO_2_ capacitors, there are also a considerable number of oxygen vacancies [39,40,41,42], which could potentially be suppressed when titanium is doped in the HfO_2_.

Although the titanium incorporation seems to suppress the leakage current, the leakage current is still relatively large. The energy band diagram [18,19] in Figure 10 attempts to provide a possible explanation in conjunction with the XPS results discussed above. From the energy band diagram in Figure 10a, titanium oxide has a relatively small band gap (3.2 eV), and the conduction band minimum is at 4.21 eV, while the band gap and conduction band minimum for germanium are 0.66 eV and 4.13 eV, respectively. The thin aluminum oxide with the thickness of about 0.3 nm is used to passivate the germanium surface and it has almost no contribution to suppressing the leakage current. If a voltage was applied at the gate on the TiO_2_/Al_2_O_3_ stack, a dramatic leakage current should be induced, considering the energy band diagram in Figure 10a. For the energy band diagram of the hafnium oxide shown in Figure 10b, the band gap is wider and the conduction band minimum is higher than that of the TiO_2_. Thus, the HfO_2_ sample has a higher potential barrier across the oxide. Therefore, the leakage current of the HfO_2_ is five times smaller than that of the TiO_2_, regardless of the deterioration of the interface caused by the oxidation of the substrate. When TiO_2_ is doped in HfO_2_, the reaction of TiO_2_ and HfO_2_ should adjust the energy band diagram as shown in Figure 10c, and the leakage current should be between that of TiO_2_ and HfO_2_ from the point view of energy band diagram. However, as mentioned above, HfO_2_ is considered to be an oxidation source and contributes to the interface deterioration, which enhances the leakage current for the HfO_2_ rich samples. Fortunately, the formation of HfTiO*_x_* in TiO_2_ doped HfO_2_ reduces the reaction between the HfO_2_ and the germanium and suppresses the deterioration of interface, which results in the significant reduction of leakage current. Therefore, in our case, the Ti_0.25_Hf_0.75_O_2_ dielectric sample has almost the same leakage current as the HfO_2_ sample while the Ti_0.9_Hf_0.1_O_2_ sample with much less HfO_2_ has the smallest leakage current among all samples.

## 3. Experimental Section

**Figure 11:**
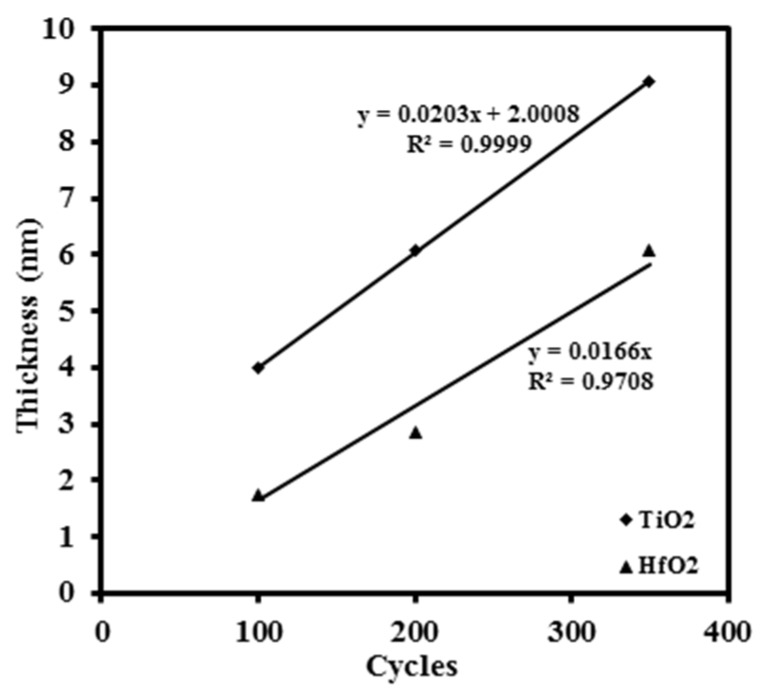
The thin film thickness *versus* ALD cycles for titanium oxide and hafnium oxide, respectively. The slopes of the two fitting straight lines (*y* = 0.0203*x* + 2.0008 and *y* = 0.0166*x*) represent the corresponding deposition rates, and R^2^ is the coefficient of determination. The deposition rates for TiO_2_ and HfO_2_ are approximately 0.203 Å/cycle and 0.166 Å/cycle, respectively.

Before mixing HfO_2_ with TiO_2_ to form Ti*_x_*Hf_1–*x*_O_2_, the ALD growth rates of TiO_2_ and HfO_2_ were tested individually. Titanium isopropoxide and methoxymethyl hafnium were used as the ALD precursors and heated to 40 °C and 100 °C, respectively. Deionized water was used as an oxygen source and argon was employed as carrier gas in all the experiments. All the deposition was performed at the substrate temperature of 300 °C. The sequence for ALD deposition was precursor pulse/purge/water pulse/purge. For both precursors, the precursor pulse duration of 3 s was followed by a purge time of 6 s. Water pulse times of 0.01 s were followed by 3 s purge times. The thickness of thin films with different ALD cycles was measured by an ellipsometer. The relationship between the film thickness and corresponding ALD cycles is shown in Figure 11. From the slopes of the fitting straight line, it was found that the deposition rates for the TiO_2_ and the HfO_2_ were approximately 0.203 Å/cycle and 0.166 Å/cycle, respectively. Based on these growth rates, the cycle ratio of the titanium oxide to the hafnium oxide was evaluated to obtain the required dielectric oxides, with the ratio of TiO_2_ to HfO_2_ being 1:3 and 9:1 (Ti_0.25_Hf_0.75_O_2_ and Ti_0.9_Hf_0.1_O_2_), respectively, in terms of thickness. For example, for Ti_0.25_Hf_0.75_O_2_, two TiO_2_ cycles (0.4 Å) were followed by seven HfO_2_ cycles (1.2 Å). The content of TiO_2_ is equal to 0.4 Å/(0.4 Å + 1.2 Å) = 25%. According to the cycle ratio and deposition rates, the total cycles for each oxide were designed to produce the required thickness of the thin films. The *p*-type germanium wafers were used as the substrates of ALD Ti*_x_*Hf_1–*x*_O_2_ thin films. The Ge wafer was cleaned ultrasonically in acetone followed by an O_2_ plasma treatment. The germanium oxide on the surface was removed by cyclically rinsing with deionized water (DI water) and diluted 2% HF. The clean wafers were transferred to the ALD chamber (Oxford Instruments OpAL™, Oxford, UK) immediately to deposit an Al_2_O_3_ passivation layer (~0.3 nm) by ALD using trimethylaluminum (TMA) as precursor. The TiO_2_, Ti_0.9_Hf_0.1_O_2_, Ti_0.25_Hf_0.75_O_2_, and HfO_2_ thin films were then deposited on the Al_2_O_3_ passivated germanium substrates, respectively. XPS measurements were carried out in a UHV system consisting of Al *K*α X-ray (1486.6 eV) source and a PSP vacuum systems 5-channel HSA electron energy analyzer. Due to an impurity in the carbon in the samples the C 1*s* peak in the spectra at 284.6 eV was used to calibrate any charging effects during measurements. The experimental XPS spectra were fitted using a Gaussian-Lorentzian line shape doublet to account for the spin-orbit splitting, using the CASAXPS fitting package. Grazing Incident X-ray diffraction (GIXRD) was carried out using a Bruker diffractometer (Bruker, Karlsruhe, Germany) with a Cu *Kα* radiation source (40 kV, 40 mA), spanning a 2θ range from 20° to 50° at a scan rate of 1°/s for all measurements. The surface morphology and roughness of the thin films were analyzed using an atomic force microscope (AFM) (Bruker, Karlsruhe, Germany). The thickness of each thin film was measured by an ELLIP-SR-1 ellipsometer with the incident angle of 65° and wavelength from 300 nm to 900 nm with a step of 20 nm. The electrode contacts with a diameter of 0.3 mm and thickness of 350 nm were deposited by E-beam evaporation (TEMD-600, Beijing, China). The back surfaces of the samples were deposited with aluminum to form ohmic contact. An Aglient 4284A precision LCR meter and a Keithley 487 picoammeter (Keithley, Cleveland, USA) were employed to investigate the electrical properties of the samples. All the electrical measurements were performed in the dark at room temperature with a Faraday Cage surrounding the prober station.

## 4. Conclusions

Hafnium titanate oxide thin films, Ti*_x_*Hf_1–*x*_O_2_, with a titanium content of *x* = 0, 0.25, 0.9, and 1 were deposited on alumina passivated germanium substrates. XPS was used to analyze the interface quality and chemical structure. The results indicated that the HfO_2_ deteriorates the interface quality, leading to an enhanced leakage current. The surface roughness was analyzed with an atomic force microscope, and all the samples exhibited relatively good surface morphology with the roughness RMS of 0.202 nm, 0.425 nm, 0.431 nm, and 0.325 nm for HfO_2_, Ti_0.25_Hf_0.75_O_2_, Ti_0.9_Hf_0.1_O_2_, and TiO_2_, respectively. XRD analysis shows that all the samples are amorphous under these deposition conditions. By using electrical characterization, it is found that there is almost no hysteresis between ramp up and ramp down of the CV curves, suggesting low trap densities. A relatively large leakage current is observed, with the lowest leakage current being about 1 mA/cm^2^ at the bias of 0.5 V for Ti_0.9_Hf_0.1_O_2_. The largest leakage current is attributed to the deterioration of the interface caused by the oxidation source borne by HfO_2_, and the small band gap of the dielectric materials.
